# Activin B Antagonizes RhoA Signaling to Stimulate Mesenchymal Morphology and Invasiveness of Clear Cell Renal Cell Carcinomas

**DOI:** 10.1371/journal.pone.0111276

**Published:** 2014-10-24

**Authors:** Ingrid Wacker, Jürgen Behrens

**Affiliations:** Experimental Medicine II, Nikolaus-Fiebiger-Center, Friedrich-Alexander Universität Erlangen-Nürnberg, Erlangen, Germany; University of Birmingham, United Kingdom

## Abstract

Activin B belongs to the TGFβ family of growth factors and is upregulated in clear cell renal cell carcinoma cells by hypoxia inducible factors. Expression of Activin B is required for tumor growth in vivo and tumor cell invasion in vitro. Here we show that activation of RhoA signaling counteracts Activin B mediated disassembly of actin stress fibers, mesenchymal cell morphology and invasiveness, whereas inhibition of RhoA rescues these effects in Activin B knockdown cells. Conversely, Activin B inhibits RhoA signaling suggesting that there is an antagonistic connection between both pathways. In addition we found that Rac1 plays an opposite role to RhoA, i.e. activation of Rac1 initiates loss of actin stress fibers, promotes a mesenchymal cell morphology and induces invasion in Activin B knockown cells, whereas inhibition of Rac1 abolishes these Activin B effects. Collectively, our data provide evidence that reduction of RhoA signaling by Activin B together with persistent Rac1 activity is a prerequisite for inducing an invasive phenotype in clear cell renal cell carcinoma.

## Introduction

Mutation of the von Hippel Lindau (VHL) tumor suppressor gene is the initial step in the development of clear cell renal cell carcinomas (ccRCC). The VHL protein functions as an E3-ubiquitin ligase targeting HIF (hypoxia inducible transcription factors) for proteasomal degradation. Hence, loss of VHL results in constitutive transcription of HIF target genes, with many of them being critically involved in tumor formation [1]–[3]. HIF directly upregulates Activin B, which is a member of the TGFβ superfamily of secreted growth factors [4]. Autocrine stimulation by Activin B evokes key features of cellular transformation in VHL-deficient cells such as a spindle shaped cell morphology, and decreased cell-matrix and cell-cell adhesion. Moreover, expression of Activin B is required for invasiveness and tumorigenicity of ccRCC cells in nude mice [4].

Activins are dimeric proteins composed of two of the four different Activin β monomers (βA, βB, βC, βE), with Activin B being a dimer of two βB subunits. Binding to specific cell-surface receptors activates Smad 2/3 dependent transcription, but also non-canonical signaling via MAP (Mitogen-activated Protein) kinases [5]. The biological outcome of Activin signaling is pleiotropic and highly dependent on the cellular context. For instance, Activins determine skin architecture and promote the re-epithelialization upon wounding [6], [7], are involved in the maintenance of pluripotency of stem cells [8], control neuronal survival and act as neuroprotective factors after ischemic brain injury [9], [10]. Despite functional redundancy between the closely related members of the Activin protein family [11], Activin B specific functions and signaling pathways have been identified, such as the induction of hyperinsulinemia by Activin B signaling via the Alk7 receptor in the pancreas [12] and the induction of anemia via activation of BMP-receptors [13]. Like TGFβ, Activins play a dual role in tumorigenesis, since they can suppress tumor cell proliferation but also support tumor growth [14]. They promote several aspects of cancer progression, such as malignant progression of skin cancer [15], development of metastases [16] and osteolytic lesions [17]. Moreover, systemically elevated Activin levels mediate cancer cachexia and Activin blocking agents are currently tested as life prolonging treatment for cancer patients [18], [19].

The protein family of small RhoGTPases cycle between an inactive, GDP-bound and an active, GTP-bound state [20], [21]. Via the activation of downstream effectors, RhoGTPases are main regulators of cytoskeleton assembly and disassembly, thereby controlling cell shape and the migratory capacity of cells. The different Rho family proteins play specific roles in this process: RhoA induces the formation of actin stress fibers [22] while Rac1 stimulates the formation of membrane ruffles and lamellipodia [23]. Since spatiotemporal coordination of Rac1 and RhoA is a prerequisite for cell migration, reciprocal regulation between both signaling pathways is common, most times with antagonistic outcome, so that RhoA inhibits Rac1 and vice versa [24]–[27]. Cytoskeletal changes enable migration and invasion of tumor cells, but are also involved in loss of epithelial cell polarity and anoikis resistance. For instance, oncogenic transformation by src or ras results in loss of actin stress fibers [28], [29] and overexpression of actin filament stabilizing proteins inhibits cellular transformation and represses tumor formation in mice [30]–[32].

Here, we show that in ccRCC cells, Activin B destabilizes actin stress fibers via downregulation of Rho pathway activity. Reduced RhoA signaling in combination with active Rac1 is required for stimulation of tumor cell invasion and induction of a spindle shaped cell morphology by Activin B.

## Materials and Methods

### Cell culture and cell treatment

786.0 [33] and RCC10 [34] were cultured in DMEM supplemented with 10% FCS and 1% penicillin/streptomycin at 37°C in a humidified atmosphere of 7.5% CO_2_. For inhibition of the RhoA pathway, cells were treated with the Rho inhibitor C3 transferase at a final concentration of 0.1 µg/ml (Cytoskeleton Inc., Denver, CO) or the Rho-Kinase inhibitor Y-27632 (Sigma-Aldrich, Steinheim, Germany) at a final concentration of 1 µM.

### Activin βB RNAi plasmids and lentiviral vectors

The pSuper-based short hairpin RNA (shRNA) expression constructs were generated by cloning annealed oligonucleotides into the BglII/HindIII-digested pSUPER vector [35]. The shRNA sequences targeting Activin βB mRNA were si1-βB 5′-GTACAACATCGTCAAGCGG-3′ and si2-βB 5′-CTTCATAGAGCAACCAGTC-3, respectively. The scrambled sequence (scr) was 5′-GGCAACATACGACTCATCT-3′. The si2-βB expression cassette was excised with XhoI and XbaI from the pSuper based Activin βB shRNA expression plasmid and cloned into the lentiviral plasmid pTRIPΔU3-EF1α/EGFP-LF [36] via XbaI/SalI.

### Lentiviral vector supernatants, viral transduction and stable knockdown of Activin βB in RCC10 cells

Lentiviral vector particles were produced as described [37]. In brief, HEK293 T cells were cotransfected with the lentiviral packaging (pCMV-dR8.2dvpr, 18 µg) and envelope (pMD.2G, 9 µg) plasmids, along with the lentiviral vector pTRIPΔU3-EF1α/EGFP-LF-shRNA (40 µg) encoding the shRNAs, in 15 cm culture dishes with polyethylenimine transfection reagent (1 mg/ml stock, 3 µl/µg DNA). Cells were washed 8 hours later. Viral supernatants were collected 48 to 60 hours after transfection, cleared by centrifugation (2000 g at room temperature) and filtered through 0.45 µm filters. An infection cocktail consisting of the retroviral supernatant and 8 µg/ml Sequabrene (Sigma-Aldrich, Steinheim, Germany) was added directly on RCC10 cells grown to 80% confluency. Infection was allowed to proceed for 24 hours, after which cells were washed and cultured further for 48 hours with fresh medium. Individual clones were selected in the presence of 2 µg/ml Puromycin and screened via quantitative PCR for efficent Activin βB knockdown.

### Expression Plasmids for RhoA and Rac1

For generation of expression constructs for RhoA and Rac1, the respective human CDS was amplified by PCR with specific oligonucleotides using 293 T cDNA as a template. Oligonucleotides used for amplification of wildtype RhoA were: RhoA_WT_frw 5′-CCCGCGGCCGCTCGCTGCCATCCGGAAGAAACTG-3′ and RhoA_rev 5′-CCCGGATCCTCACAAGACAAGGCACCCAGATT-3′. Oligonucleotides used for amplification of RhoAG14V were: RhoA_G14V_frw 5′-CCCGCGGCCGCTCGCTGCCATCCGGAAGAAACTGGTGATTGTTGGTGATGTAGCC-3′ and and RhoA_rev. Oligonucleotides used for amplification of RhoAT19N were: RhoA_T19N_frw 5′-CCCGCGGCCGCTCGCTGCCATCCGGAAGAAACTGGTGATTGTTGGTGATGGAGCCTGTGGAAAGAACTGC-3′ and RhoA_rev. Oligonucleotides used for amplification of wildtype Rac1 were: Rac1_WT_frw 5′-CCCGCGGCCGCTCCAGGCCATCAAGTGTGTGG-3′ and Rac1_rev 5′-CCCGGATCCTTACAACAGCAGGCATTTTCTCTTC-3′. Oligonucleotides used for amplification of Rac1G12V were: Rac1_G12V_frw 5′-CCCGCGGCCGCTCCAGGCCATCAAGTGTGTGGTGGTGGGAGACGTAGCT-3′ and Rac1_ rev. Oligonucleotides used for amplification of Rac1_T17N were: Rac1_T17N_frw 5′-CCCGCGGCCGCTCCAGGCCATCAAGTGTGTGGTGGTGGGAGACGTAGCT-3′ and Rac1_rev. PCR products were cloned via NotI/BamHI into pEGFP-C3-Not and the correct sequence of each plasmid was verified.

### Generation of stable transfectants for RhoA and Rac1

400.000 cells were plated onto 10 cm dishes and transfected with 10 µg of the respective RhoA or Rac1 expression construct using Polythylenimin as transfection reagent. 2 µg pBabe was cotransfected for selection purposes. Individual clones were selected in the presence of 2 µg/ml puromycin and analyzed for GFP expression via fluorescence microscopy. To generate pools, equal cell numbers of 6 individual transfectants were combined.

### Invasion assay

Calf skin type I collagen G (Serva electrophoresis GmbH, Heidelberg, Germany) and Rat tail type I collagen R (Biochrom AG, Berlin, Germany) were mixed at a ratio of 1∶1, 0.1 vol of sodium bicarbonate (22 mg/ml) and 0.1 vol of 10**×**DMEM was added, and the solution was neutralized with sodium hydroxide. Aliquots of 1.2 ml/well were allowed to gel in 6-well culture dishes at 37°C. 5**×**10^4^ tumor cells were seeded in DMEM containing 2% FCS onto the collagen surfaces. Cells invading the collagen I gel were detected by focusing down into the matrix using a 20x objective, and quantified after 3 days by counting 30 randomly chosen optical fields per well [38].

### Western Blotting

Subconfluent cells grown in 3 wells of a 6-well culture dish were scraped into 100 µl lysis buffer (135 mM Tris/HCl pH 6.8, 75 mM NaCl, 2.5 mM EDTA, 10% Glycerol, 10% β-Mercaptoethanol, 4% SDS, 0.5% Triton-X 100, 1 mM DTT, 0.2% Bromophenol Blue, 50 mM NaF) and lysed with an ultrasonic homogenizer. Lysates were snap-frozen in liquid nitrogen. Equal amounts of protein were separated by 10% SDS-PAGE and proteins were transferred to HybondN nitrocellulose membranes (Amersham, Freiburg, Germany). Antibodies against human phosphorylated MLC2 (Cell Signaling Technology inc., Danvers, MA), total human MLC2 (Cell Signaling Technology inc., Danvers, MA; clone D18E2), human β-actin (Cell Signaling Technology inc., Danvers, MA, clone 13E5), GFP (Roche Diagnostics, Mannheim, Germany; clone 7.1 and 13.1), as well as peroxidase-conjugated secondary antibodies (Jackson ImmunoResearch, Cambridgeshire, UK) were used according to the manufacturer’s instructions. Signals were visualized using Enhanced Chemoluminescence Reagent (Perkin Elmer, Rodgau, Germany) and detected with a luminescent image analyser (LAS-3000, Fuji, Düsseldorf, Germany). Densitometric analysis was performed using Aida software.

### Staining of the actin cytoskeleton

Cells were seeded on glass coverslips, fixed at a subconfluent state with 3% paraformaldehyde for 15 min, and permeabilized with 0.5% Triton. After blocking with 10% FCS in DMEM, F-actin (filamentous actin) was stained using tetramethylrhodamine isothiocyanate labeled-phalloidin (Sigma-Aldrich, Steinheim, Germany). Cell micrographs were obtained with a CCD camera (Visitron, Munich, Germany) on a Zeiss Axioplan 2 imaging microscope (Zeiss, Oberkochen, Germany).

### G-lisa assay

The HRP-linked immunosorbent assay (ELISA)-based G-LISA kit (Cytoskeleton Inc., Denver, CO) was used to determine endogenously active RhoA levels in 786.0 parental cells and RCC10 parental cells, Activin B knockdown and control clones according to the manufacturer’s instructions. In brief, 25.000 cells were seeded in 6-well plates. 16 hours after seeding, cells were cultured for 24 hours with DMEM supplemented with 1% FCS. Subsequently, cells were lysed in 100 µl of G-LISA lysis buffer per well and snap-frozen in liquid nitrogen. For the G-LISA assay, cell lysates were thawed in a room-temperature water bath, and equal protein amounts were added to a 96-well dish coated with the RhoA binding domain of RhoA effector proteins and incubated at 4°C for 30 min under vigorous shaking. Active RhoA levels were determined by subsequent incubations with anti-RhoA antibody (1∶500) and secondary horseradish peroxidase-conjugated antibody (1∶2000) for 45 min each under vigorous shaking at room temperature. After adding the HRP detection reagent, luminescence was immediately detected by a microplate luminescence reader. For determination of active RhoA, luminescence obtained for cell lysates was corrected for background luminescence obtained with lysis buffer (Relative Light Units). For each lysate luminescence was determined in triplicates.

### Proliferation assay

Proliferation was determined by WST-1 cell proliferation reagent (Roche Diagnostics, Mannheim, Germany) according to the manufacturer’s instructions. In brief, 1000 cells per well were seeded in a 96-well plate in DMEM containing either 10%, 2% FCS or 2% FCS supplemented with 1 µM Y-27632. The number of viable cells was determined 14, 38, 62 hours after seeding by incubation of cells with 100 µl DMEM containing 10 µl of the tetrazolium salt WST-1 for 2 hours. The amount of WST-1 cleaved to formazan was quantified by measurement of the absorbance at 450 nm using an ELISA reader. The absorbance at 690 nm was determined as a reference wavelength. Triplicates were used for each condition.

### RNA isolation and reverse transcription PCR (RT-PCR)

Total RNA was isolated with the Qiagen RNeasy Kit (QIAGEN, Hilden, Germany) and contaminating genomic DNA was enzymatically removed with DNase I (QIAGEN, Hilden, Germany). cDNA was generated with the AffinityScript QPCR cDNA Synthesis Kit (Agilent technologies, Boeblingen, Germany) according to the manufactures instructions. 500 ng RNA were used as template in a 20 µl reverse transcription reaction containing 10 µl first strand master mix (2×), 3 µl Random Primers (0.1 µg/µl) and 1.0 µl of AffinityScript RT/RNase Block enzyme mixture. Primer annealing occured for 5 min at 25°C, followed by 45 min at 42°C for cDNA synthesis. The reaction was terminated at 95°C for 5 min. Quantitative PCR was performed with 1 µl cDNA as a template and KAPA SYBR FAST universal mastermix (PEQLAB Biotechnologie GmbH, Erlangen, Germany) using the iCycler (Biorad, Hercules, CA, USA) detection system according to the manufacturer’s instructions. Oligonucleotides used for amplification of Activin βB cDNA: inhbb_frw 5′-TGCTCTAGACCACCGTGCCTTGCACTG-3′ and inhbb_rev 5′-TCTCTCCGACTGACAGGCATTTG-3′. Oligonucleotides used for amplification of β-Actin cDNA: Actin-s 5′-AGTCCTGTGGCATCCACGAAA-3′ and Actin-as 5′-GTCATACTCCTGCTTGCTGA-3′. Primer annealing occurred at 58°C for 20 sec.

### Statistical analysis

Statistical analysis of two groups was performed by a paired student’s t-test. Statistical analysis of multiple groups was performed with one-way ANOVA and subsequent Tuckey’s honest significance as post-hoc test. Significance was denoted by asterisks: *p<0.05; **p<0.0.1; **p<0.001; n.s. not significance.

## Results

### Activin B dependent invasion of ccRCC cells is inhibited by serum

**Figure 1:**
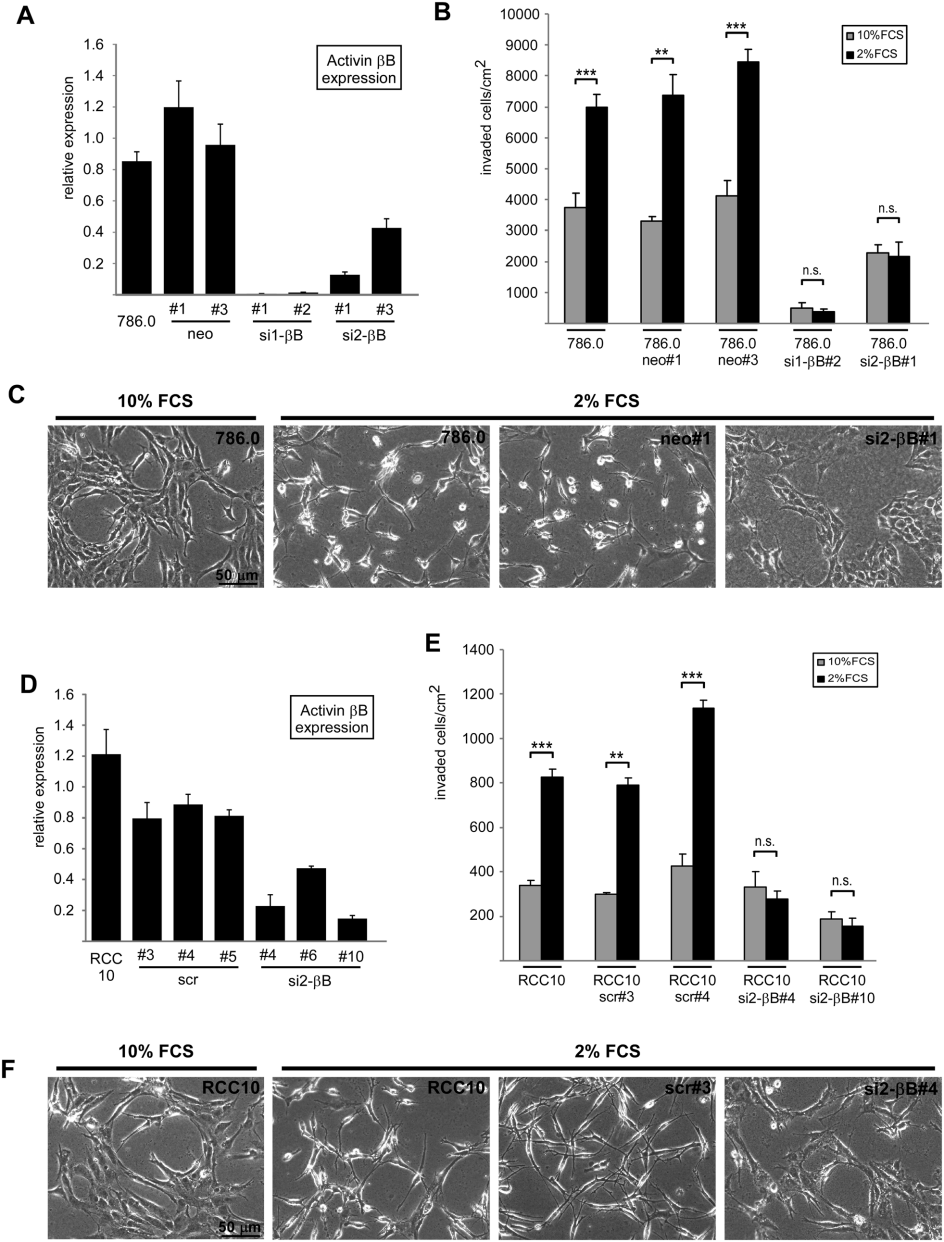
Activin B dependent invasion of ccRCC cells is inhibited by serum. (**A**) Relative Activin B expression of 786.0 parental tumor cells and individual clones carrying empty vector (neo#1, #3) and two different shRNAs targeting Activin B mRNA (si1-βB#1 and #2, si2-βB#1 and #3), respectively, determined by quantitative realtime RT-PCR. Results were normalized on β-actin expression. (**B**) Invasion of parental 786.0 tumor cells, neo control clones and Activin B knockdown clones plated on collagen I gels in the presence of 2% (black bars) and 10% FCS (grey bars). Number of invaded cells was quantified after 3 days. (**C**) Phase contrast micrographs corresponding to B depicting the morphology of the indicated cell lines on the surface of the collagen I gel. (**D**) Relative Activin B expression of RCC10 parental tumor cells and individual clones carrying scrambled shRNA (scr#3, #4, #5) and shRNA targeting Activin B mRNA (si2-βB#4, #6, #10), respectively, determined by quantitative realtime RT-PCR. Results were normalized on β-actin expression. (**E**) Invasion of parental RCC10 tumor cells, scrambled control clones and Activin B knockdown clones plated on collagen I gels in the presence of 2% (black bars) and 10% FCS (grey bars). Number of invaded cells was quantified after 3 days. (**F**) Phase contrast micrographs corresponding to E depicting the morphology of the indicated cell lines on the surface of the collagen I gel. Bars in A, B, D and E represent the mean of three independent experiments, error bars indicate standard deviation. Statistical significance was determined by Student’s t-test and denoted by asterisks: **P<0.01; ***P<0.001; n.s. not significant.

In this study we used 786.0 and RCC10 ccRCC cell lines, which exhibit high levels of Activin B as a consequence of VHL-deficiency. To specifically interfere with Activin B expression we made use of Activin B knockdown clones of 786.0 cells previously generated in our laboratory [4], and established new Activin B knockdown clones of the RCC10 cell line. Quantitative RT-PCR confirmed strong knockdown of Activin B (Figure 1A, D).

When plated on collagen I gels, parental 786.0 and RCC10 cells efficiently invaded the gel which was inhibited by knockdown of Activin B as previously reported [4]. When optimizing the assay, we noticed that decreasing serum concentration from 10% to 2% strongly increased invasion of the parental cells and control clones which was blocked by knockdown of Activin B (Figure 1B, E, black bars versus grey bars). The drastic decrease in invaded cells upon Activin B knockdown is not due to altered cell division, since proliferation assays revealed only minor clonal differences that did not correlate with Activin B expression levels (Figure S1). We also observed morphological changes induced by low serum: in the presence of 10% FCS 786.0 and RCC10 cells formed cell cluster with flattened and well spread cytoplasm and an epithelial-like phenotype, whereas in the presence of 2% FCS, they acquired a spindle shaped mesenchymal-like cell morphology with multiple thin protrusions and reduced contacts to neighboring cells. This shift in cell morphology at low serum is dependent on Activin B as shown by Activin B knockdown cells, which phenotypically resembled cells at 10% FCS (Figure 1C, F). Thus, Activin B dependent invasiveness and concomitant morphological changes are enhanced by low serum conditions indicating that serum counteracts Activin B activity.

### Knockdown of Activin B stabilizes actin stress fibers in ccRCC cells

**Figure 2:**
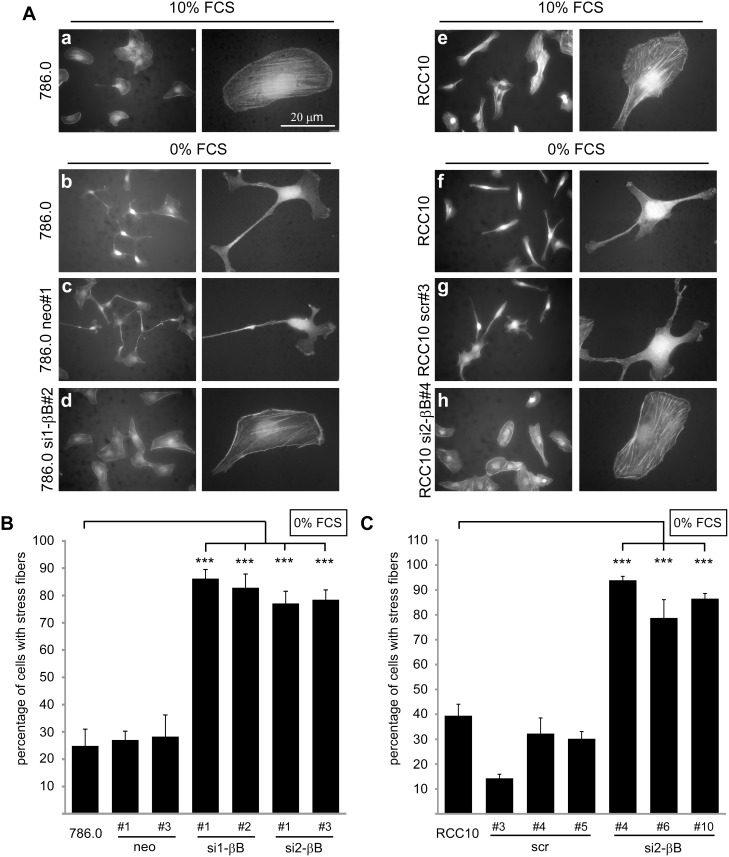
Knockdown of Activin B stabilizes actin stress fibers in ccRCC cells. (**A**) Subconfluent parental 786.0 and RCC10 cells, control clones (786.0 neo#1, RCC10 scr#3) and Activin B knockdown clones (786.0 si1-βB#2, RCC10 si2-βB#4), respectively, were maintained in the presence of 10% FCS or serum starved (0% FCS) for 3 hours. Micrographs show TRITC-labeled Phalloidin staining of the actin cytoskeleton. (**B**) and (**C**) Quantification of the indicated 786.0 (**B**) and RCC10 cells **(C)** with actin stress fibers upon serum starvation for 3 hours. Phalloidin stained cells were classified by microscopic analysis and at least 200 cells were counted per experiment. Bars represent the mean of four independent experiments, error bars indicate standard deviation. Statistical significance was determined by ANOVA analysis and denoted by asterisks: ***P<0.001.

The drastic changes in cell shape induced by Activin B may include reorganization of the cytoskeleton. To investigate this, we visualized the actin cytoskeleton with TRITC-labeled phalloidin. In the presence of 10% FCS both 786.0 and RCC10 cells appeared spread-out and actin was organized in stress fibers (Figure 2A, a, e). Upon serum starvation for 3 hours (0% FCS), parental cells and control clones became spindle shaped and almost completely lost actin stress fibers, frequently leading to accumulation of actin in the tips of cell protrusion (Figure 2A, b, c, f, g). In contrast, the majority of Activin B knockdown clones maintained stress fibers and a flattened and well spread cytoplasm (Figure 2A, d, h) suggesting that Activin B induces changes in the cytoskeleton under low serum conditions. Quantification of these results is shown in Figure 2B, C and Figure S2.

### Knockdown of Activin B activates the RhoA pathway

**Figure 3:**
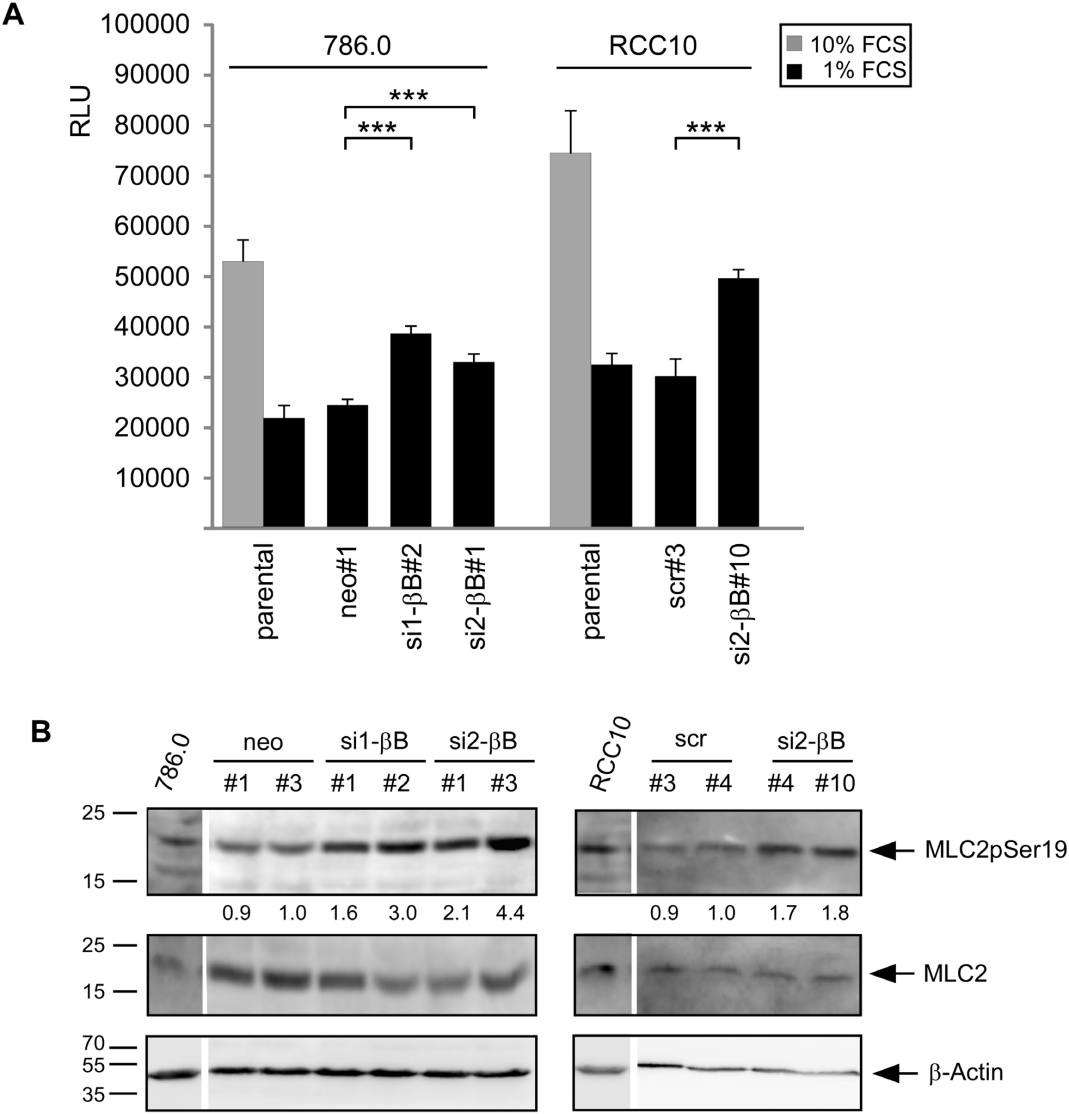
Knockdown of Activin B activates the RhoA pathway. (**A**) Endogenous RhoA activity of the indicated cell lines determined by G-Lisa assay. Bars represent the mean of three independent experiments, error bars indicate standard deviation. Statistical significance was determined by ANOVA analysis and denoted by asterisks; ***P<0.001. (**B**) Western Blot analysis of Myosin Light Chain 2 (MLC2) phosphorylated at Serin 19 in the indicated cell lines upon serum starvation (0% FCS) for 4 hours. pMLC2 was detected by a phospho specific antibody. Total MLC2 and β-actin served as loading controls. Numbers below the lanes reflect relative levels of phosphorylated MLC2 normalized to total MLC2 as determined by densitometry.

Our results suggests that serum induces signaling events that counteract Activin B mediated changes in cell invasion and the actin cytoskeleton. One possible candidate to mediate theses effects is RhoA, which is crucial for stress fiber formation and activated by serum [22], [39]. To decipher the interplay between Activin B and RhoA signaling we analyzed whether Activin B alters RhoA activity in 786.0 and RCC10 cells. In the presence of 10% FCS both cell lines showed high RhoA activity. In contrast, in the presence of 1% FCS, RhoA activity was strongly reduced in parental and control clones, but remained significantly higher in Activin B knockdown clones (Figure 3A). Thus, high Activin B signaling inhibits RhoA activity under serum starvation. To confirm this, we chose phosphorylation of the Rho-Kinase target Myosin light chain 2 (MLC2) as a second readout of RhoA activity [40]. Knockdown of Activin B increased MLC2 phosphorylation in both 786.0 and RCC10 cells, in line with a role of Activin B in inhibiting RhoA signaling (Figure 3B).

### The RhoA pathway is required for actin stress fiber formation induced by Activin B knockdown

**Figure 4:**
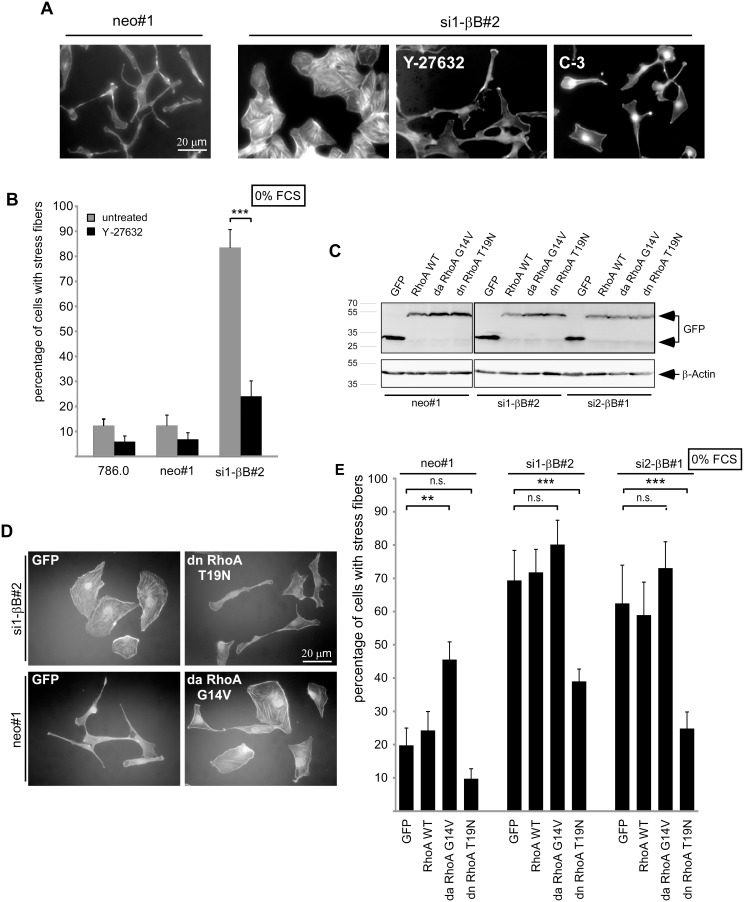
The RhoA pathway is required for actin stress fiber formation induced by Activin B knockdown. (**A**) TRITC-labeled Phalloidin staining of serum starved (0% FCS) neo#1 control cells and si1-βB#2 Activin B knockdown cells either untreated or treated with a Rho-Kinase inhibitor (Y-27632) or a Rho inhibitor (C3-Exoenzyme). (**B**) Quantification of untreated (grey bars) and Y-27632 treated (black bars) cells with actin stress fibers. (**C**) Generation of pools of 786.0 neo#1 and Activin B knockdown clones (si1-βB#2, si2-βB#1) stably transfected with EGFP empty vector, expression constructs for GFP-tagged wildtype RhoA, dominant active RhoA (G14V) or dominant negative RhoA (T19N). Expression of EGFP and GFP-tagged RhoA proteins was confirmed by Western Blotting. (**D**) TRITC-labeled Phalloidin staining of the indicated pools after serum starvation (0% FCS) for 3 hours. (**E**) Quantification of cells with actin stress fibers upon serum starvation for 3 hours. Bars represent the mean of three (B) or five (E) independent experiments, error bars indicate standard deviation. Statistical significance was determined by Student’s t-test (4B) and ANOVA analysis (4E) and denoted by asterisks: **P<0.01; ***P<0.001; n.s. not significant.

If Activin B acts via suppression of RhoA, cellular changes induced by loss of Activin B should be rescued by interfering with RhoA signaling. Indeed, inhibition of the Rho effector Rho-Kinase by the inhibitor Y-27632, or of RhoA by cell permeable C-3 exoenzyme abolished stress fiber formation in 786.0 Activin B knockdown clones and changed their morphology towards a spindle like appearance resembling those of Activin B expressing cells (neo#1) (Figure 4A, B). Similarly, expression of dominant negative RhoA in Activin B knockdown cells (Figure 4C) led to spindle-shaped cells that have lost stress fibers (Figure 4D, E). Dominant negative RhoA also increased the number of spindle shaped 786.0 cells in the presence of 10% FCS (Figure S3). Conversely, aberrant RhoA activation by dominant active RhoA in Activin B expressing neo#1 control cells (Figure 4C) reduced the number of spindle shaped cells and promoted stress fiber formation, confirming that RhoA acts antagonistically to Activin B (Figure 4D, E). Collectively, these data show that inhibition of RhoA signaling triggers morphological changes that are similar to those induced by activation of Activin B signaling, and activation of RhoA signaling prevented Activin B effects. Together with the known regulation of RhoA by serum this suggests that serum antagonizes Activin B induced effects via activation of the RhoA pathway.

### The RhoA pathway interferes with invasiveness of ccRCC cells

**Figure 5:**
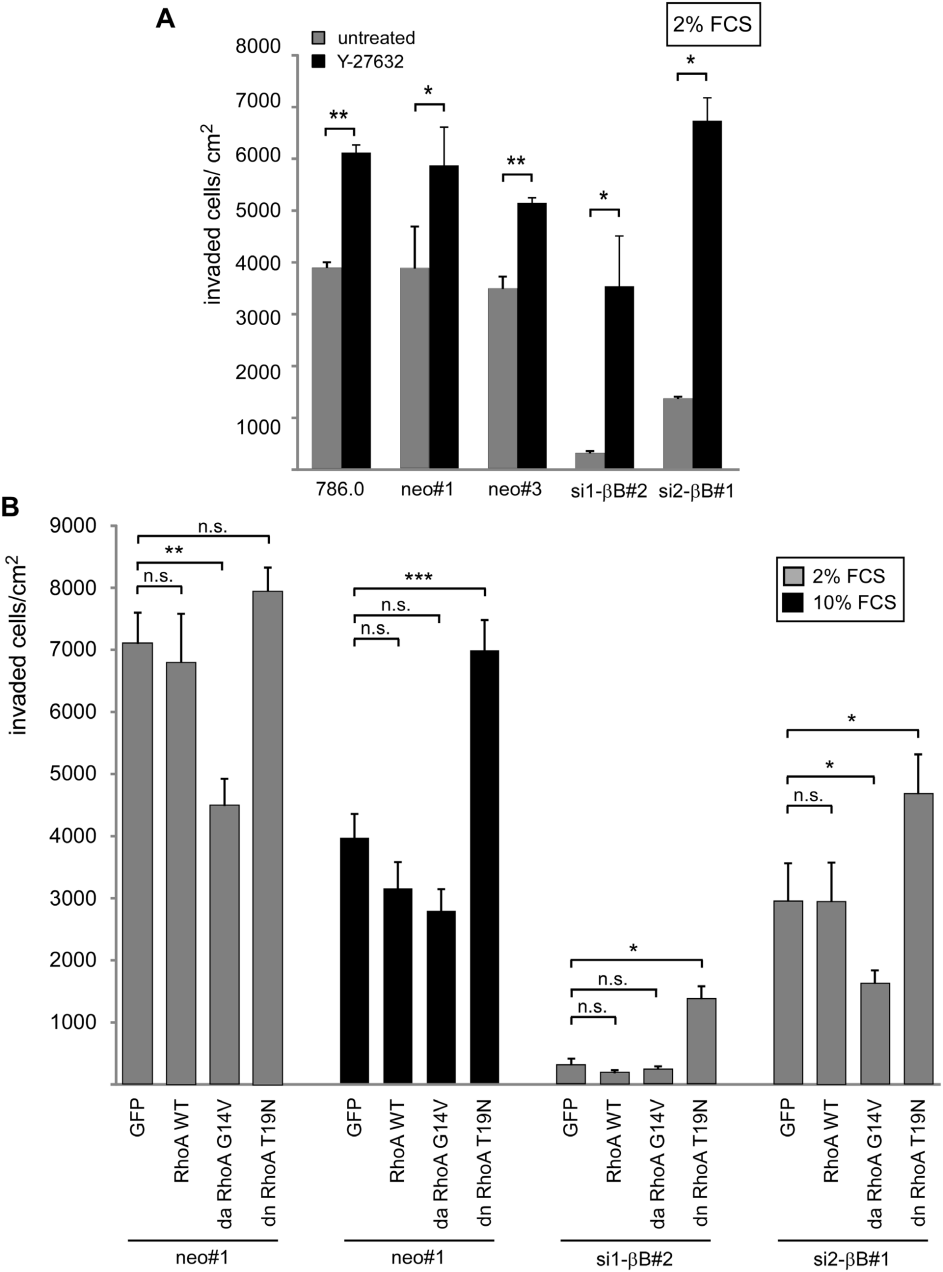
The RhoA pathway interferes with invasiveness of ccRCC cells. (**A**) Parental 786.0, neo control clones and Activin B knockdown clones were plated in the presence of 2% FCS on collagen I gels and treated with the Rho-Kinase inhibitor Y-27632 (black bars) or left untreated (grey bars). Number of invaded cells was quantified after 3 days. (**B**) Stable pools expressing either EGFP, wildtype, dominant active (G14V) and dominant negative (T19N) RhoA, respectively, were plated in the presence of 2% FCS (grey bars) or 10% FCS (black bars) on collagen I gels. Invaded cells were quantified after 3 days. Bars represent the mean of three independent experiments, error bars indicate standard deviation. Statistical significance was determined by Student’s t-test (5A) and ANOVA analysis (5B) and denoted by asterisks: *P<0.05; **P<0.01; ***P<0.001; n.s. not significant.

To address the role of the RhoA pathway in Activin B mediated invasiveness, we altered RhoA activity in collagen I invasion assays. Inhibition of Rho-Kinase with Y-27632 stimulated invasiveness of Activin B expressing but also Activin B knockdown clones (Figure 5A). Expression of dominant negative RhoA also increased invasiveness of Activin B knockdown cells showing that RhoA suppresses invasiveness. Conversely, dominant active RhoA significantly diminished invasiveness of Activin B expressing neo#1 cells and further reduced the remaining invasiveness of the si2-βB#1 knockdown clone (Figure 5B). Neither chemical inhibition of Rho-Kinase nor expression of the different RhoA proteins had any effect on cell proliferation, thus the observed changes in the number of cells within the collagen I matrix must be due to altered invasiveness (Figure S4A, B). These differences in invasion were accompanied by changes in cell morphology of the RhoA transfectants on collagen I gels. Dominant active RhoA generated epithelial-like cell clusters in Activin B expressing cells, whereas dominant negative RhoA led to dissociated spindle-like cells in Activin B knockdown cells (Figure S5). Altogether, these loss and gain of function experiments demonstrate that RhoA signaling is active in ccRCC cells and counteracts invasiveness. Activin B becomes ineffective in stimulating invasiveness when RhoA is activated either by serum or a constitutive active RhoA mutant, and vice versa RhoA inhibition induces invasiveness in the absence of Activin B. The repression of invasion by high serum as shown in in Figure 1B and E is most likely mediated by RhoA because serum could no longer repress the invasive capacity of cells expressing dominant negative RhoA (Figure 5B, black bars).

### Rac1 and RhoA have opposite roles in Activin B dependent actin rearrangement and invasion

**Figure 6:**
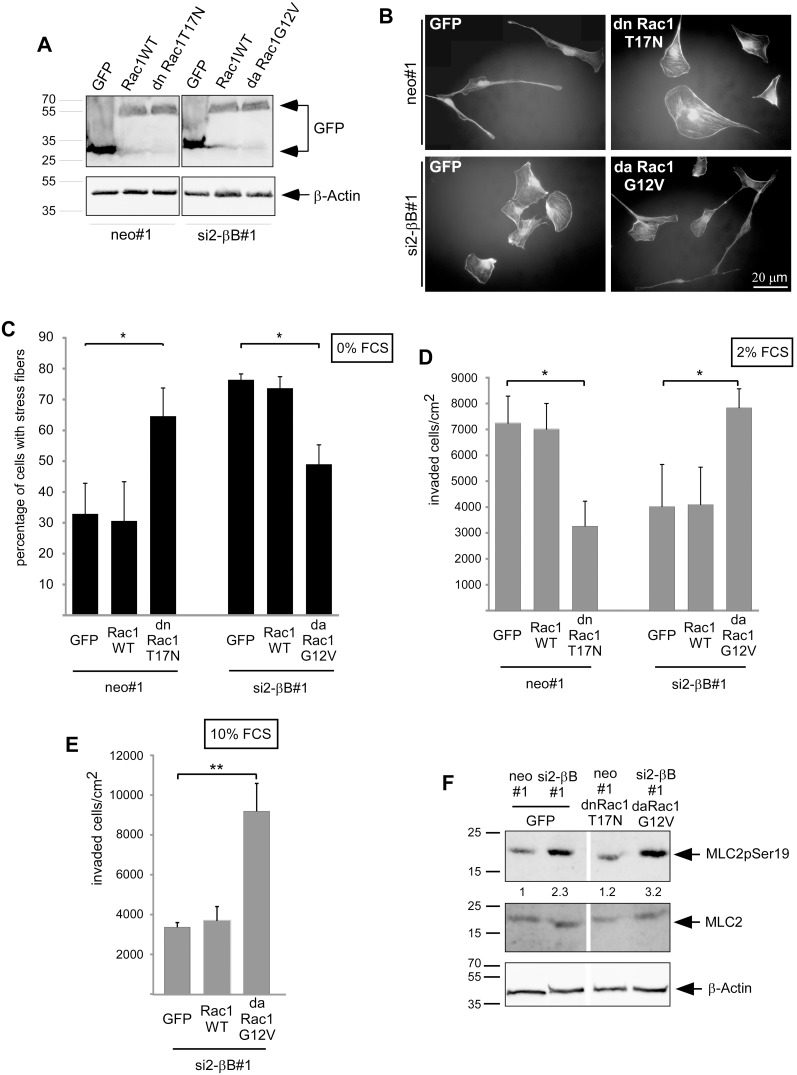
Opposite roles for Rac1 and RhoA in Activin B dependent invasion and actin rearrangement. (**A**) Generation of pools of the neo#1 clone and of the si2-βB#1 Activin B knockdown clone stably transfected with EGFP empty vector, expression constructs for GFP-tagged wildtype Rac1, dominant active Rac1 (G12V) or dominant negative Rac1 (T17N). Expression of EGFP and GFP-tagged Rac1 proteins was confirmed by Western Blotting. (**B**) TRITC-labeled Phalloidin staining of the indicated pools after serum starvation (0% FCS) for 3 hours. (**C**) Quantification of cells with actin stress fibers upon serum starvation for 3 hours. (**D**) and (**E**) The indicated pools were plated in the presence of 2% FCS (**D**) or 10% FCS (**E**) on collagen I gels and the number of invaded cells was quantified after 3 days. (**F**) Phosphorylation of MLC2 (Myosin light chain 2) at Serin 19 in 786.0 pools expressing either EGFP or the indicated Rac1 proteins was determined by Western Blotting. Cells were serum starved (0% FCS) for 4 hours. pMLC2 was detected by a phospho specific antibody. Total MLC2 and β-actin served as loading controls. Numbers below the lanes reflect relative levels of phosphorylated MLC2 normalized to total MLC2 as determined by densitometry. Bars in C, D and E represent the mean of three (C) and four (D; E) independent experiments, error bars indicate standard deviation. Statistical significance was determined by ANOVA analysis and denoted by asterisks: *P<0.05; **P<0.01.

Besides RhoA, Rac1 is also involved in the control of cell migration, and the ratio between RhoA and Rac1 activity determines the mode of cell migration as either amoeboid or mesenchymal [27]. We therefore analyzed whether Rac1 is involved in Activin B triggered changes in the cytoskeleton, cell morphology and invasion. For this purpose, we established pools of stable transfectants expressing either EGFP, GFP-tagged wildtype Rac1, dominant active Rac1 (Rac1G12V) or dominant negative Rac1 (Rac1T17N) in the neo#1 control clone or the si2-βB#1 Activin B knockdown clone of 786.0 cells (Figure 6A). Phalloidin staining of the actin cytoskeleton revealed that dominant negative Rac1 increased the number of cells exhibiting stress fibers in the Activin B expressing neo#1 clone. Conversely, dominant active Rac1 decreased the percentage of cells containing stress fibers in the Activin B knockdown clone (Figure 6B, C). On collagen I gels, dominant negative Rac1 strongly decreased invasiveness and induced epithelial-like clusters of spread-out cells in the Activin B expressing neo#1 clone (Figure 6D; Figure S6). Vice versa, dominant active Rac1 increased the number of Activin B knockdown cells invading into the collagen I gel up to the level of neo#1 cells (Figure 6D). This enhanced invasiveness correlated with the loss of cell-cell contacts and the development of a mesenchymal-like phenotype (). Also under conditions of high RhoA signaling, i.e. in the presence of 10% FCS, cells expressing activated Rac1 gained invasiveness (Figure 6E). The expression of the different Rac1 proteins had no effect on cell proliferation, thus the observed changes in the number of cells within the collagen I matrix must be due to altered invasiveness (Figure S4C). Thus, in ccRCC cells inhibition of Rac1 generates phenotypes similar to knockdown of Activin B or activation of RhoA, and activation of Rac1 has the opposite effects. This suggests that Rac1 is involved in mediating Activin B effects and that it acts oppositely to RhoA. To gain insight into the hierarchy of Rac1 and RhoA in 786.0 cells, we analyzed MLC2 phosphorylation as readout for RhoA activity in the established pools of Rac1 transfectants. Dominant negative Rac1 did not increase the low MLC2 phosphorylation in Activin B expressing neo#1 cells, and dominant active did not suppress high MLC2 phosphorylation in the Activin B knockdown cells (Figure 6F). Thus, Rac1 might act downstream or independently of RhoA.

## Discussion

**Figure 7:**
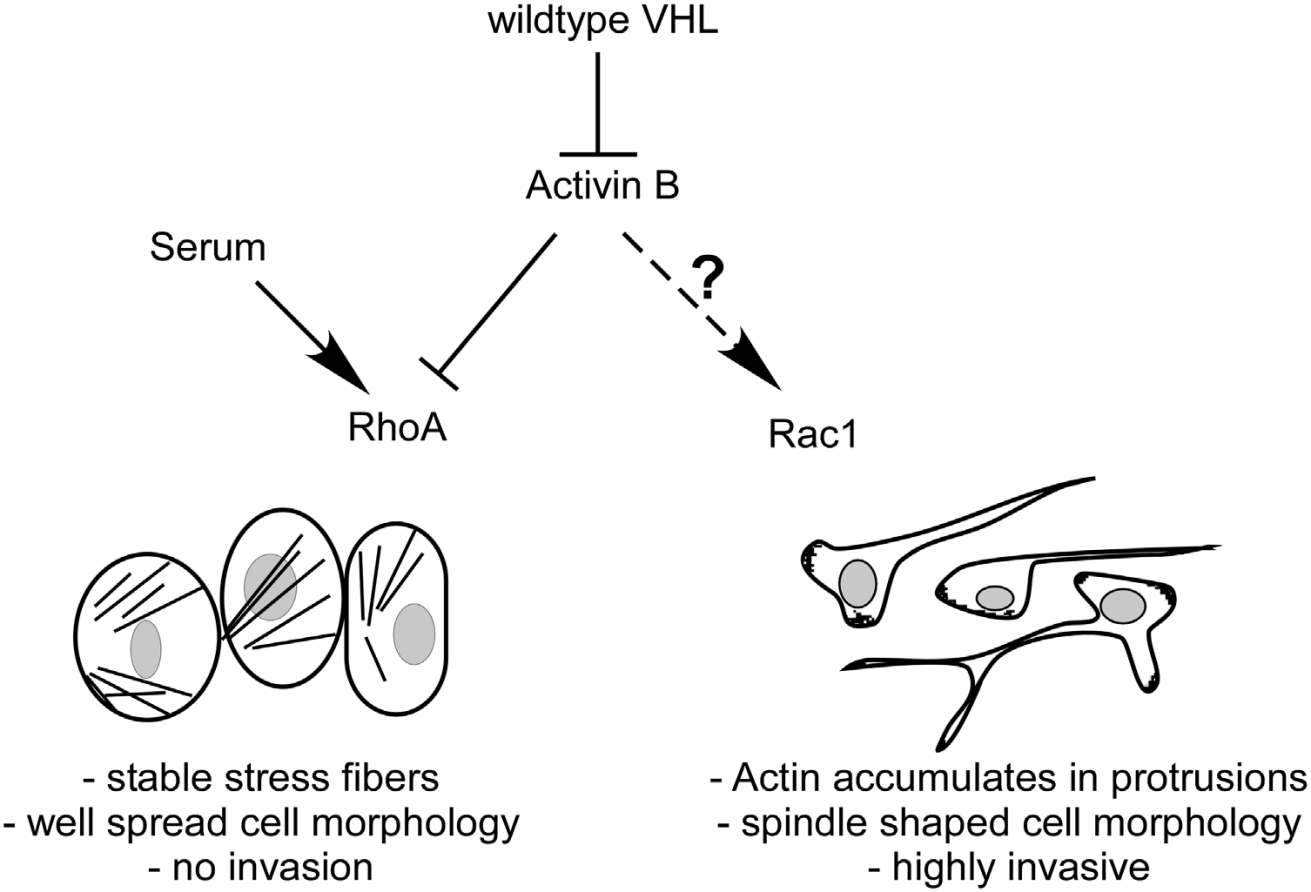
Model. Autocrine stimulation of VHL negative ccRCC tumor cells by Activin B reduces RhoA activity thereby reorganizing the actin cytoskeleton and inducing invasiveness of spindle-shaped cells. Serum, which promotes RhoA activity, counteracts Activin B mediated effects. Rac1 activation by an unknown mechanism plays an opposite role to RhoA and is required for the invasive phenotype.

We have previously discovered Activin B as a target gene of HIF that is upregulated in renal cell carcinomas as a consequence of VHL mutation. Activin B induces cell invasion and morphological alterations towards a mesenchymal phenotype including loss of actin stress fiber formation. We now show that Activin B and RhoA signaling have opposing roles in these processes in renal cell carcinoma cells. We found that Activin B effects are counteracted by serum and show that RhoA activity underlies these serum effects. Conversely, Activin B inhibits RhoA activity. Thus, a balance between Activin B and RhoA signaling determines functional outcome towards either the invasive or non-invasive phenotype of ccRCC. Furthermore, Rac1 is shown to act antagonistic to RhoA in this pathway. Our data provides a first hint that in ccRCC cells Rac1 might act downstream of RhoA, though further work is required to clarify the interplay between RhoA and Rac1 on endogenous level. So far, it is also not clear whether under conditions of reduced serum concentrations, Activin B directly promotes Rac1 activation or whether cooperation with other factors is needed (Figure 7).

Our data are based on our initial finding that increasing serum concentrations blocks key events controlled by Activin B, namely cell invasion, mesenchymal cell shape and actin rearrangements. We chose RhoA as a possible candidate to mediate these effects as it promotes actin stress fiber formation and is activated by serum. We propose that at low serum concentrations RhoA signaling is active at a constitutive basal level whereas at higher serum concentration its activity is increased shifting the balance towards inhibition of Activin B effects. RhoA signaling also prevails at low serum when Activin B is downregulated as in our Activin B knockdown clones.

The morphological alterations induced by Activin B resemble those seen in epithelial-mesenchymal transitions (EMT), which are linked to invasiveness of epithelial cells. However, expression of E-cadherin and the EMT inducing transcription factors snail and slug were not altered Activin B expression (I. Wacker unpublished observations) suggesting that the standard EMT program is not induced by Activin B. Notably, cell scattering and induction of invasiveness by Rho-Kinase inhibitor in MCF7 breast tumor cells was shown to be similarly independent of EMT [41]. It seems that the antagonistic interplay between Activin B and RhoA signaling determines the phenotypic balance between an epithelial-like, low invasive and a mesenchymal, high invasive state via changing the actin cytoskeleton.

In our experimental system we used knockdown of Activin B to dissect its function and we found that the degree of knockdown correlates well with functional outcome (see for instance Figure 1, 5). We noticed that treatment of knockdown cells with recombinant Activin B did not immediately rescue effects induced by Activin B knockdown but required a long term treatment of several days, finally leading to cells that were no longer dependent on exogenous factor addition (I.W. unpublished data). This suggests that Activin B exposure leads to a gradual reprogramming of the cells towards an invasive phenotype. This reprogramming might involve epigenetic alterations induced by Activin B. The situation is reminiscent of long-term TGFβ treatment of ras transformed epithelial cells which leads to a stable fibroblastic phenotype independent of further TGFβ treatment. In this experimental system cells were shown to produce TGFβ in an autocrine fashion making them independent of exogenous TGFβ [42], [43]. In the continuous presence of shRNA to Activin B a similar autocrine induction of Activin B is unlikely to cause the stable reversion of the knockdown ccRCC cells, yet it is possible that cells produce other factors such as TGFβ or Activin A that substitute for Activin B. It is currently unclear how Activin B and RhoA signaling are connected on a molecular level.

The ratio of RhoA and Rac1 activities determines the mode of cell migration [27]. Cells with high RhoA and low Rac1 activity were shown to move as round cells in an amoeboid way whereas cells with low RhoA and high Rac1 activity move as elongated cells in a mesenchymal fashion [27], [44], [45]. Our data suggest that migration of ccRCC cells follows the latter mechanism as inhibition of Rac1 abolished the spindle-like mesenchymal cell morphology of Activin B expressing cells and inhibited their invasion, whereas activation of Rac1 sufficed for re-establishing mesenchymal morphology and invasiveness in Activin B knockdown cells. As dominant negative Rac1 did not affect the phosphorylation level of MLC2, we speculate that it acts downstream of or in parallel to RhoA in ccRCC cells. Activin B signaling might shift the balance between Rac1 and RhoA towards high Rac1 activity.

It is becoming increasingly clear that the tumor microenvironment plays key roles in epithelial transformation processes including control of tumor stem cells and cellular invasion and metastasis. It is tempting to speculate that by altering RhoA signaling, e.g. through activation of extracellular pathways, the tumor microenvironment might determine whether or not Activin B promotes invasion of ccRCC cells [41]. Of note, our previous data showed that Activin B expression can be induced in other tumor cell lines than ccRCC by exposure to low oxygen, which represents the physiological activation mechanism of HIF. It is therefore possible that our findings are relevant to cancers beyond renal cell carcinoma.

## Supporting Information

Figure S1 **(A)** and **(B)** Proliferation of parental tumor cells, control clones and Activin B knockdown clones in the presence of 2% FCS was determined by WST-1 assay over a period of three days. The graphs show relative absorbance at 450 nm corrected for absorbance at 690 nm. **(A)** 786.0 cells, **(B)** RCC10 cells.
(PDF)

Figure S2 Quantification of the indicated 786.0 and RCC10 cells with actin stress fibers in the presence of 10% FCS. Phalloidin stained cells were classified by microscopic analysis.
(PDF)

Figure S3 **(A)** Relative Activin βB expression of neo control and Activin B knockdown cells (si1-βB#2, si2-βB#1) stably transfected with either EGFP or the indicated GFP tagged RhoA proteins determined by quantitative realtime PCR. β-actin was used for normalization. **(B)** The indicated pools were cultured in the presence of 10% FCS and the percentage of cells with stress fibers was quantified by microscopic analysis of Phalloidin stained cells.
(PDF)

Figure S4 **(A)** Proliferation of stable pools expressing either EGFP, wildtype, dominant active (G14V) and dominant negative RhoA (T19N), respectively, in the presence of 2% FCS was determined by WST-1 assay over a period of three days. The graphs show relative absorbance at 450 nm corrected for absorbance at 690 nm. **(B)** Proliferation of neo#1 control clone and si1-βB#2 Activin B knockdown clone in the presence of the Rho-Kinase inhibitor Y-27632, respectively. **(C)** Proliferation of stable pools expressing either EGFP, wildtype, dominant active (G12V) and dominant negative Rac1 (T17N), respectively, was determined in the presence of 2% FCS.
(PDF)

Figure S5 **(A)** and **(B)** Cell morphology of stable pools expressing either EGFP, dominant active (G14V) and dominant negative (T19N) RhoA, respectively, plated on collagen I gels. **(A)** 2% FCS. Note the induction of cell clusters by dominant active RhoA (G14V) in the neo control clone (boxed) and the induction of spindle shaped cells by dominant negative RhoA (T19N) in the Activin B knockdown clone (arrows). **(B)** 10% FCS. Note the spindle shaped morphology of cells expressing dominant negative RhoA (T19N) despite the presence of high serum.
(PDF)

Figure S6 **(A)** Relative Activin βB expression of neo control and Activin B knockdown cells stably transfected with either EGFP or the indicated GFP tagged Rac1 proteins determined by quantitative realtime PCR. β-actin was used for normalization. **(B)** Cell morphology of the indicated pools plated on collagen I gels. Note the induction of cell clusters by dominant negative Rac1 (T17N) and the induction of spindle shaped cells by dominant active Rac1 (G12V).
(PDF)
