# Supplementary figures and images for: Activin B Antagonizes RhoA Signaling to Stimulate Mesenchymal Morphology and Invasiveness of Clear Cell Renal Cell Carcinomas
Source: PLoS One. 2014 Oct 24;9(10):e111276. doi: 10.1371/journal.pone.0111276 (PMC4208853; doi:10.1371/journal.pone.0111276)

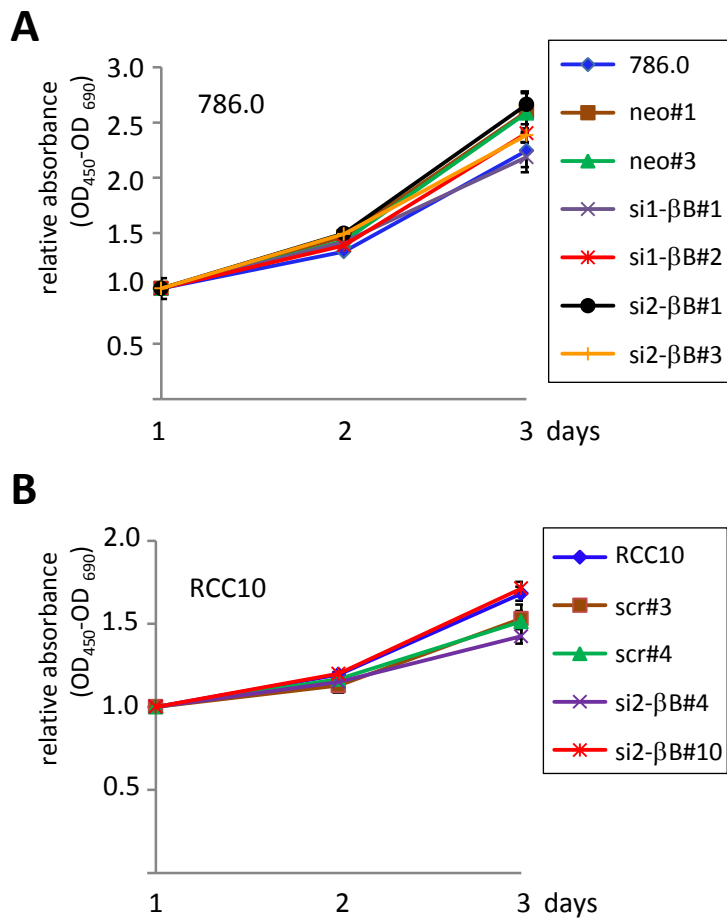

**Figure S1**

Supplement: Figure S1 — (A) and (B) Proliferation of parental tumor cells, control clones and Activin B knockdown clones in the presence of 2% FCS was determined by WST-1 assay over a period of three days. The graphs show relative absorbance at 450 nm corrected for absorbance at 690 nm. (A) 786.0 cells, (B) RCC10 cells. (PDF) [file pone.0111276.s001.pdf]

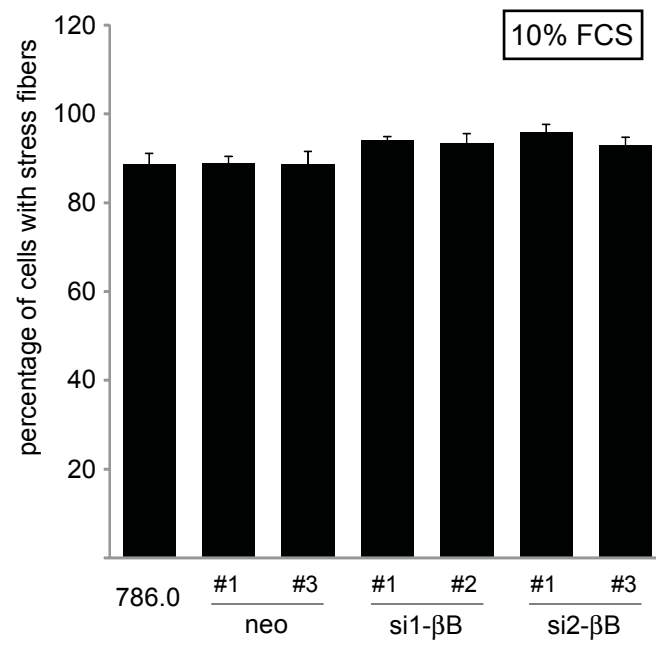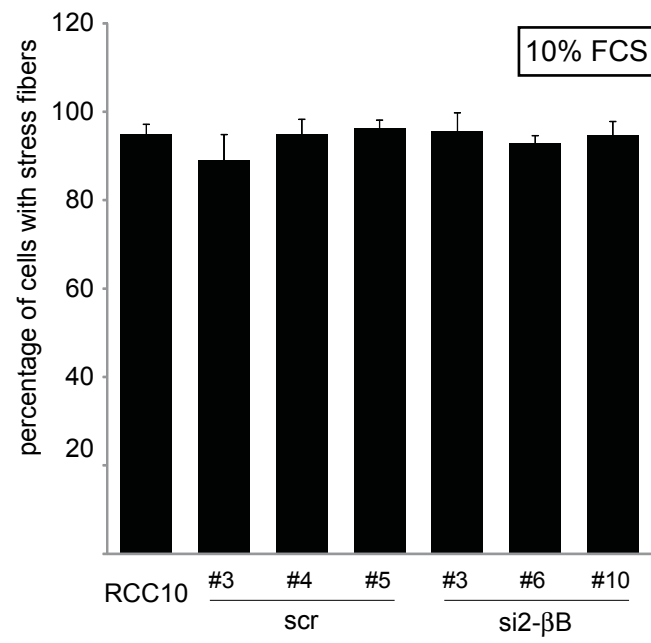

**Figure S2**

Supplement: Figure S2 — Quantification of the indicated 786.0 and RCC10 cells with actin stress fibers in the presence of 10% FCS. Phalloidin stained cells were classified by microscopic analysis. (PDF) [file pone.0111276.s002.pdf]

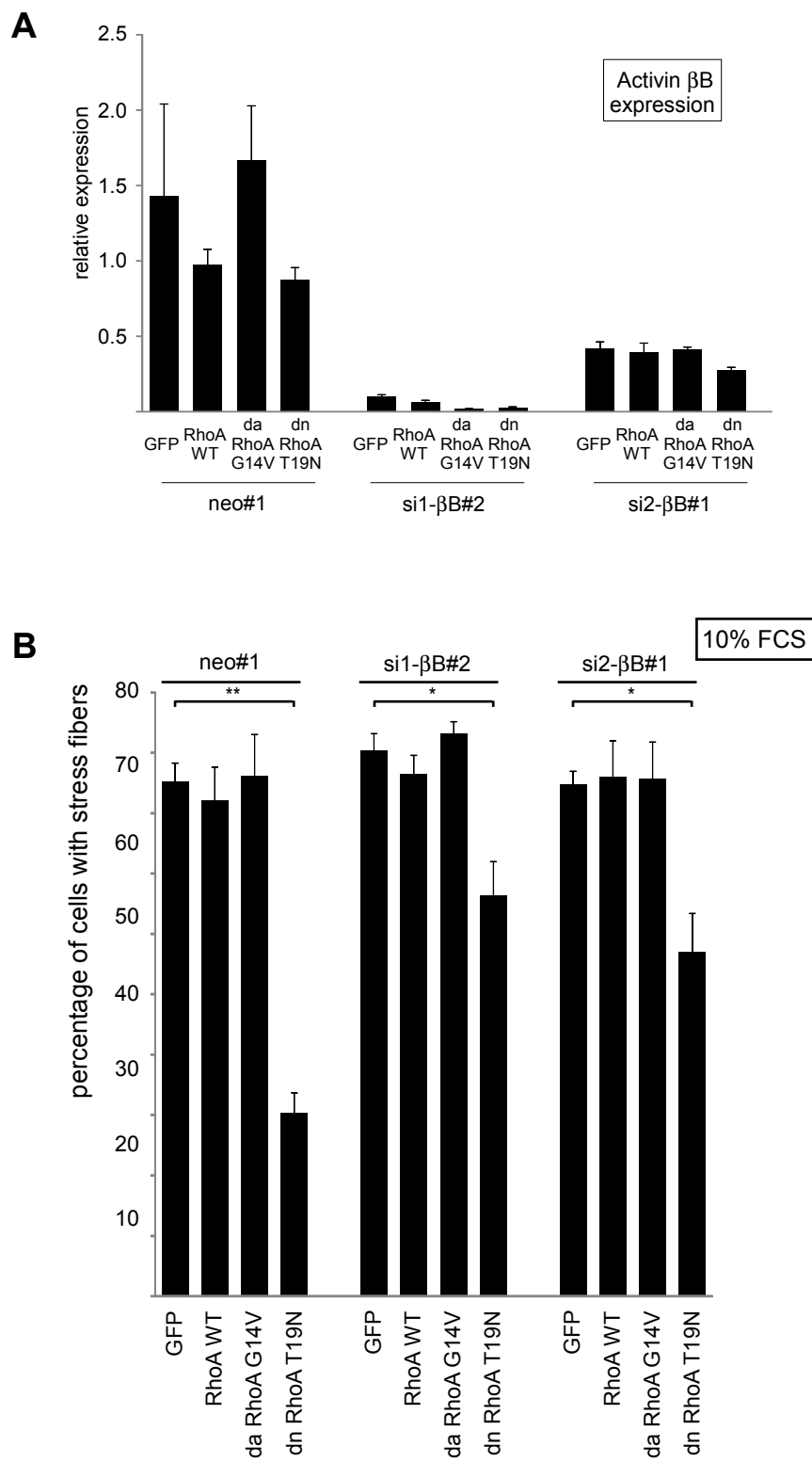

**Figure S3**

Supplement: Figure S3 — (A) Relative Activin βB expression of neo control and Activin B knockdown cells (si1-βB#2, si2-βB#1) stably transfected with either EGFP or the indicated GFP tagged RhoA proteins determined by quantitative realtime PCR. β-actin was used for normalization. (B) The indicated pools were cultured in the presence of 10% FCS and the percentage of cells with stress fibers was quantified by microscopic analysis of Phalloidin stained cells. (PDF) [file pone.0111276.s003.pdf]

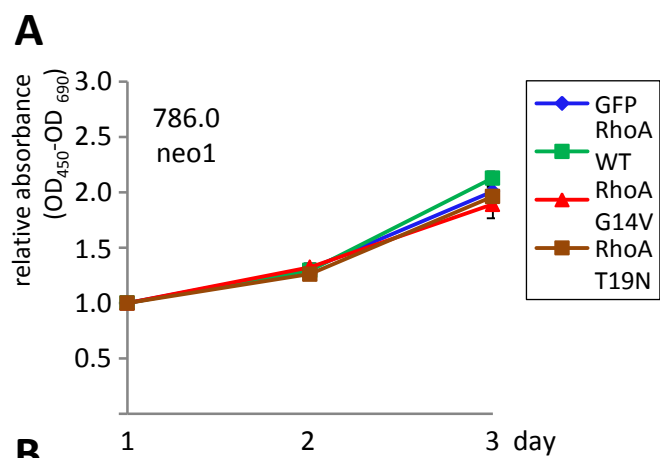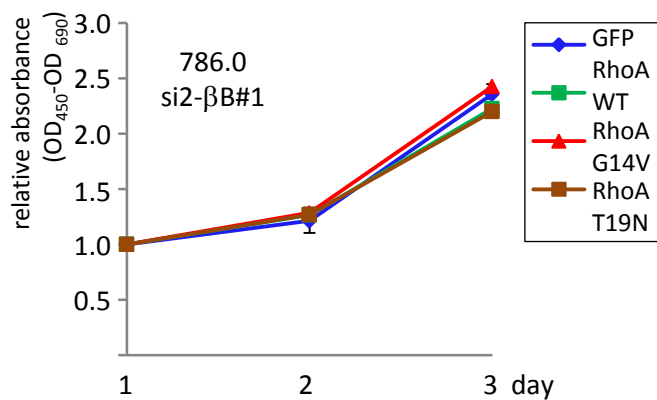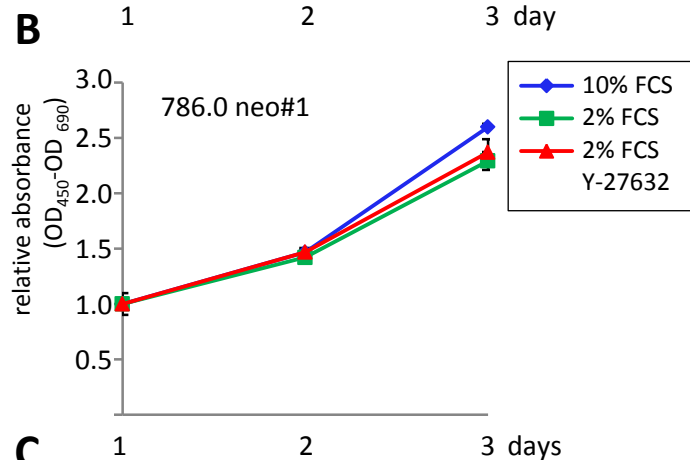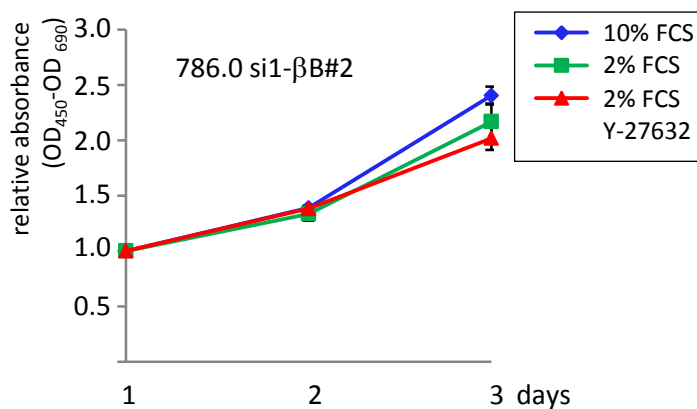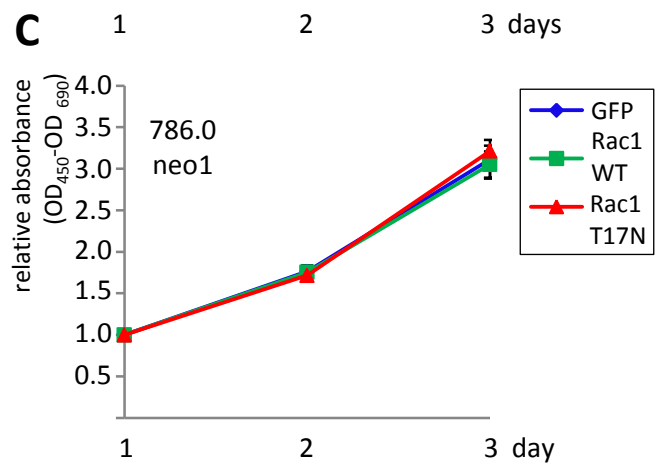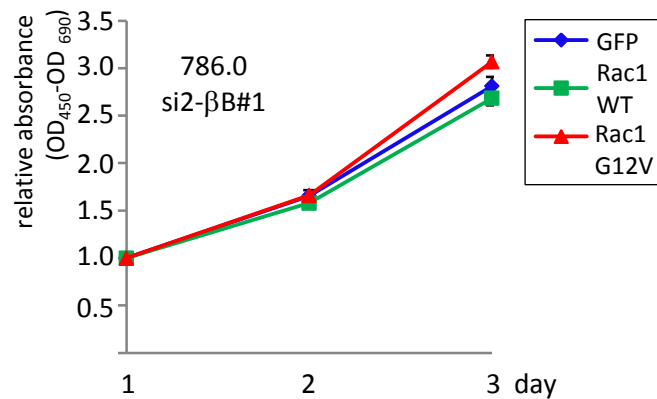

**Figure S4**

Supplement: Figure S4 — (A) Proliferation of stable pools expressing either EGFP, wildtype, dominant active (G14V) and dominant negative RhoA (T19N), respectively, in the presence of 2% FCS was determined by WST-1 assay over a period of three days. The graphs show relative absorbance at 450 nm corrected for absorbance at 690 nm. (B) Proliferation of neo#1 control clone and si1-βB#2 Activin B knockdown clone in the presence of the Rho-Kinase inhibitor Y-27632, respectively. (C) Proliferation of stable pools expressing either EGFP, wildtype, dominant active (G12V) and dominant negative Rac1 (T17N), respectively, was determined in the presence of 2% FCS. (PDF) [file pone.0111276.s004.pdf]

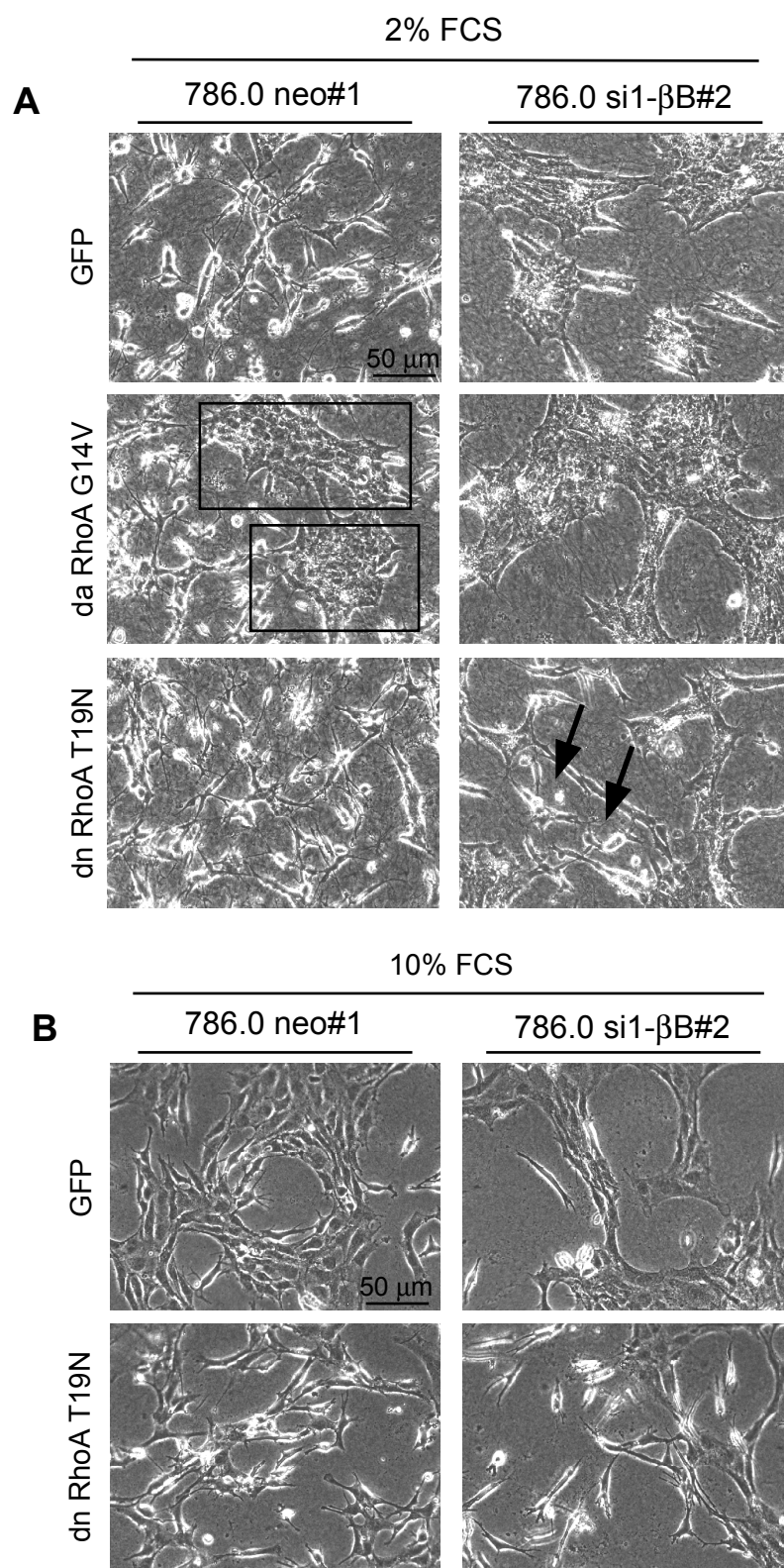

**Figure S5**

Supplement: Figure S5 — (A) and (B) Cell morphology of stable pools expressing either EGFP, dominant active (G14V) and dominant negative (T19N) RhoA, respectively, plated on collagen I gels. (A) 2% FCS. Note the induction of cell clusters by dominant active RhoA (G14V) in the neo control clone (boxed) and the induction of spindle shaped cells by dominant negative RhoA (T19N) in the Activin B knockdown clone (arrows). (B) 10% FCS. Note the spindle shaped morphology of cells expressing dominant negative RhoA (T19N) despite the presence of high serum. (PDF) [file pone.0111276.s005.pdf]

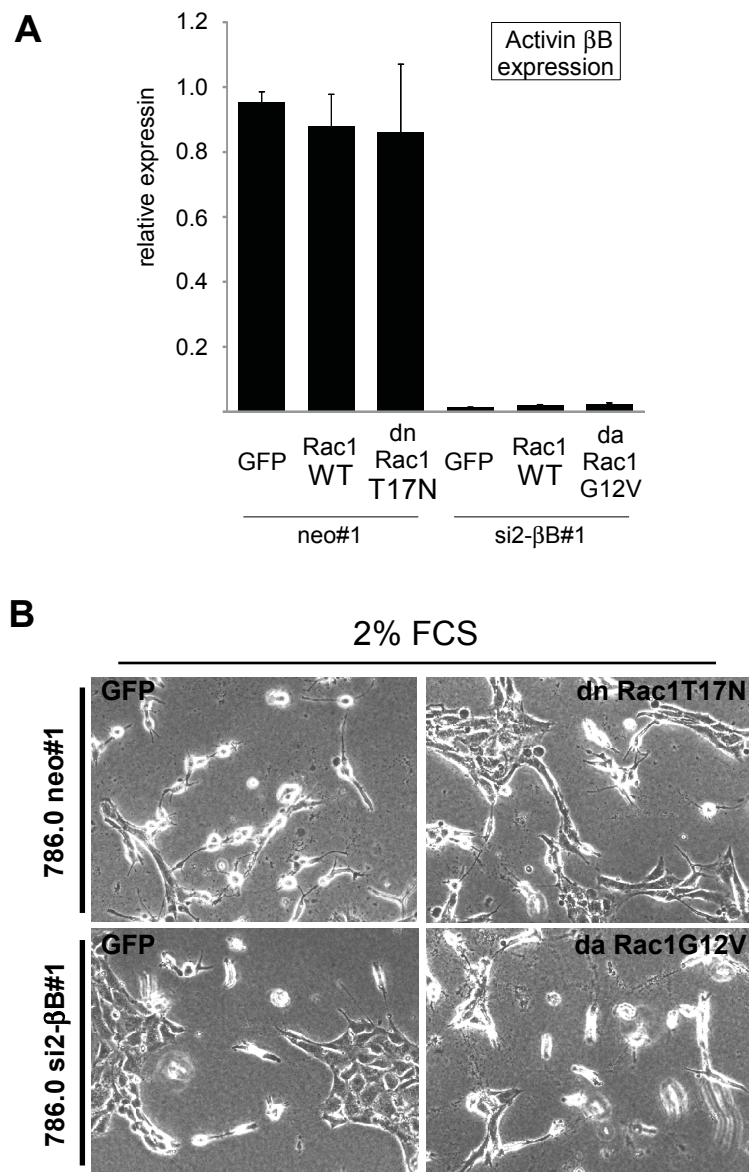

**Figure S6**

Supplement: Figure S6 — (A) Relative Activin βB expression of neo control and Activin B knockdown cells stably transfected with either EGFP or the indicated GFP tagged Rac1 proteins determined by quantitative realtime PCR. β-actin was used for normalization. (B) Cell morphology of the indicated pools plated on collagen I gels. Note the induction of cell clusters by dominant negative Rac1 (T17N) and the induction of spindle shaped cells by dominant active Rac1 (G12V). (PDF) [file pone.0111276.s006.pdf]
